# Editorial: Enhancing innate immunity in combination therapy for solid tumors

**DOI:** 10.3389/fimmu.2024.1539027

**Published:** 2025-01-03

**Authors:** David Pejoski

**Affiliations:** ^1^ Adoram Therapeutics, Geneva, Switzerland; ^2^ School of Pharmaceutical Sciences, University of Geneva, Geneva, Switzerland; ^3^ Institute of Pharmaceutical Sciences of Western Switzerland, University of Geneva, Geneva, Switzerland

**Keywords:** innate immunity, combination therapy, immuno-oncology, solid tumor, immunotherapy, cancer

Solid tumors often consist of an immunosuppressive tumor microenvironment (TME), and higher levels of cellular and genetic heterogeneity compared to hematological tumors (1). Primary and metastatic solid tumor sites may also be challenging to detect and access via surgical or therapeutic means, despite recent advances in the field (2). These aspects of solid tumors provide a rationale for the use of combination therapies to increase clinical response rates, for example, by leveraging synergistic therapeutic mechanisms (3), or overcoming the cellular and genetic heterogeneity that contributes to resistance to monotherapies.

One element that impacts patient prognosis is the innate immune cell milieu within solid tumors, for example, the presence and activation status of natural killer (NK) cells, macrophages, innate lymphoid cells (ILCs) and dendritic cells (DCs) - reviewed in (4). Hence, modulating the innate immune system via a wide array of drug targets and modalities, has emerged as an attractive immunotherapeutic approach. Some essential mechanisms by which the innate immune system contributes to tumor rejection include augmenting soluble or membrane bound factors that promote tumor rejection, which can enhance infiltration, trafficking, or activation of anti-tumor immune cells. The anti-tumor effects may be mediated by the innate cells themselves, by priming the adaptive system, or modifying stromal or endothelial cells in a way that favors tumor rejection.

In this Research Topic entitled ‘*Enhancing Innate Immunity in Combination Therapy for Solid Tumors’*, six articles contribute to the body of knowledge from a molecular, cellular, therapeutic, and clinical perspective. Boersma et al. defines a specific role for the innate immune system in cancer progression, and Ota et al. showcases the ability to harness toll-like receptor-targeting immunostimulants in combination therapies. The additional four reviews, hypotheses, or opinion pieces provide a current overview of the potential application of CAR-NK cell radiotherapy combinations (He et al.), effects of enterosorbents on innate immunity (Shichkin et al.), current research landscape of combination therapies in non-small cell lung cancer (NSCLC) (Wu et al.), and role of tissue-resident macrophages (TRMs) in breast cancer pathogenesis and therapy (Biswas).

At the molecular level, modulation of even a single protein within the innate immune system can have profound effects on cancer progression and resolution. This was exemplified in this Research Topic by gasdermin D (Boersma et al.), which forms a cytokine-releasing pore that mediates pyroptosis. Furthermore, TLR7, a pathogen recognition receptor that can be modulated with therapeutic agents such as DSP-0509 (Ota et al.), shows promise as a therapeutic target, with over 380 TLR-7 targeting clinical trials initiated thus far, including drug combinations with a variety of other immunotherapies - notably other immunomodulatory agents including molecular adjuvants and immune checkpoint blockade antibodies (5). If we consider PDL1 as a clinically validated innate immune drug target, there is ample support for exploring combination therapies including this pathway. This is evidenced by the variety of clinically-approved (6) or experimental attempts (7), and the fact that 83% of recent clinical trials featuring anti-PDL1 immune checkpoint blockade (ICB) are in combination with other drugs rather than as a single agent (8). Conversely, modulating the balance of many innate immunity pathways simultaneously was explored in the opinion article on enterosorption (Shichkin et al.). This approach involves the oral administration of enterosorbents to remove multiple soluble immunomodulators from the digestive tract, which are known or speculated to promote tumor growth, invasion, and metastasis.

Within the diverse army of anti-tumor innate immune cell subsets, NK cells are a major contributor to immunosurveillance and tumor cell clearance. The success of CAR-T cell therapy thus inspired similar approaches with innate immune cell subsets (9), including CAR-modified NK cells (10) – with the promise of an improved safety and manufacturing profile, as described in (He et al). To date, over 160 clinical trials have been initiated to evaluate CAR-NK immunotherapies. To improve efficacy, preclinical studies of CAR-NK cell therapy in combination with radiotherapies have recently been reported (He et al.). The rationale for this combination pair is that radiotherapy exposes tumor cell DNA, which results in endothelial adhesion and cytokine-mediated activation of NK cells. Additional studies highlight the promise of combining CAR-NK with other immunomodulatory drugs, for example Nutlin3a (11), in order to improve outcomes when applied to various solid tumor types that harbor a dysfunctional p53.

NSCLC and breast cancer are two of the most prevalent cancer types, and are thus the subject of intense combination therapy research that aims to overcome the limitations of monotherapy, such as immune-evasion or T cell exhaustion (12). The NSCLC therapy review (Wu et al.) delves into the main classes of approved and experimental drug combinations, with selected examples of innate-targeting approaches, such as IL-6 and IL-12 cytokine therapy. Since cytokines can be produced by innate cells to polarize the TME and the ensuing adaptive response, modulating the cellular source of cytokines is also being pursued in the clinic. For example, tissue-resident macrophages (TRMs) in breast cancer, are known to produce tumor-promoting cytokines such as TGF-ß, IL-10, and CCL-8, but could be targeted therapeutically (‘reprogrammed’) to secrete cytokines that promote tumor rejection instead (Biswas). Globally, the range of innate immune-associated drug targets is vast, comprising of distinct secreted and cell surface marker signatures that impact on both immune and non-immune components of solid tumors (13). Above all, this demonstrates the complexity of the TME which calls for precise genetic, phenotypic, and ontogenic characterizations to provide a basis from which improved therapies can be designed. With an increasing understanding of innate immune cell signaling pathways, drug developers have an improved ability to design rational combination therapies that elicit the desired effects, which can be different within and across solid cancer types.

To conclude, innate immunity-based strategies remain a rapidly emerging frontier, predicted to make significant contributions to the solid tumor treatment landscape. By modulating innate immunity, including key cellular and soluble effectors mechanisms, novel pathways are being unlocked to complement conventional and existing immunotherapeutic cancer treatment regimens. The current Research Topic offers summaries of some of the emerging paradigms in fundamental and applied immuno-oncology, as well as new insights into innate immune molecular mechanisms that could be harnessed to favor effective tumor eradication. I sincerely thank the authors of the articles, in addition to the dedicated co-editors and peer reviewers for their contributions.
